# Hybrid Three-Dimensional Spiral WSe_2_ Plasmonic Structures for Highly Efficient Second-Order Nonlinear Parametric Processes

**DOI:** 10.1155/2018/4164029

**Published:** 2018-12-09

**Authors:** Xianqing Lin, Yingying Liu, Kang Wang, Xiaolong Liu, Yongli Yan, Yong Jun Li, Jiannian Yao, Yong Sheng Zhao

**Affiliations:** ^1^Key Laboratory of Photochemistry, Institute of Chemistry, Chinese Academy of Sciences, Beijing 100190, China; ^2^University of Chinese Academy of Sciences, Beijing 100049, China

## Abstract

Two-dimensional (2D) layered materials, with large second-order nonlinear susceptibility, are currently growing as an ideal candidate for fulfilling tunable nanoscale coherent light through the second-order nonlinear optical parametric processes. However, the atomic thickness of 2D layered materials leads to poor field confinement and weak light-matter interaction at nanoscale, resulting in low nonlinear conversion efficiency. Here, hybrid three-dimensional (3D) spiral WSe_2_ plasmonic structures are fabricated for highly efficient second harmonic generation (SHG) and sum-frequency generation (SFG) based on the enhanced light-matter interaction in hybrid plasmonic structures. The 3D spiral WSe_2_, with AA lattice stacking, exhibits efficient SH radiation due to the constructive interference of nonlinear polarization between the neighboring atomic layers. Thus, extremely high external SHG conversion efficiency (about 2.437×10^−5^) is achieved. Moreover, the ease of phase-matching condition combined with the enhanced light-matter interaction in hybrid plasmonic structure brings about efficient SHG and SFG simultaneously. These results would provide enlightenment for the construction of typical structures for efficient nonlinear processes.

## 1. Introduction

Broadband tunable coherent light sources with small-footprint and low power consumption have attracted great attention because of their potential applications ranging from high-throughput sensing to on-chip photonic communication [1–4]. Second harmonic generation (SHG) and sum-frequency generation (SFG) [5, 6], which are based on the second-order nonlinear optical parametric processes, are emerging as ideal alternatives to nanoscale lasers due to their wide wavelength modulation range. Transition metal dichalcogenides (TMDC), with broken inversion symmetry structure in the monolayer limit which brings about nonvanishing second-order nonlinear susceptibility [7–9], have been widely applied for obtaining SHG at nanoscale size. Unfortunately, the atomic thickness of TMDC leads to weak light-matter interaction owing to the nearly 1 nm interaction length. Meanwhile, due to the diffraction limit of light, the small sizes (at subwavelength scale) of TMDC lead to poor field confinement, which results in low nonlinear optical conversion efficiency. Thus, thicker TMDC nanostructures with effective subwavelength electromagnetic field confinement are needed for highly efficient nonlinear optical applications.

In this work, we demonstrate the construction of hybrid 3D spiral TMDC plasmonic structures for highly efficient second-order nonlinear parametric processes by combining the large second-order nonlinear susceptibility (on the order of 10^−7^ m/V) of 3D spiral TMDC materials with the high subwavelength confinement of electric field of surface plasmonic polaritons (SPP). The 3D spiral TMDC structures, where the basal planes shrink gradually to the top while spiraling up, exhibit efficient SH radiation due to the broken inversion symmetry structure with AA lattice stacking. Meanwhile, the increase of light-matter interaction length due to the increasing atomic layers, along with the highly concentrated local field in hybrid plasmonic structure, leads to the great improved nonlinear optical conversion efficiency (2.437×10^−5^), which is higher than most other reported metal-related nanostructures. Furthermore, benefitting from the ease of phase-matching condition and enhanced light-matter interaction in hybrid plasmonic structure, the SHG and SFG are realized simultaneously when the hybrid 3D spiral TMDC plasmonic structures were excited by two separated fundamental waves (FWs). We believe that the results demonstrated here would provide guidance for the development of nonlinear optical devices with high conversion efficiency and specific functionalities.

## 2. Results

**Figure 1:**
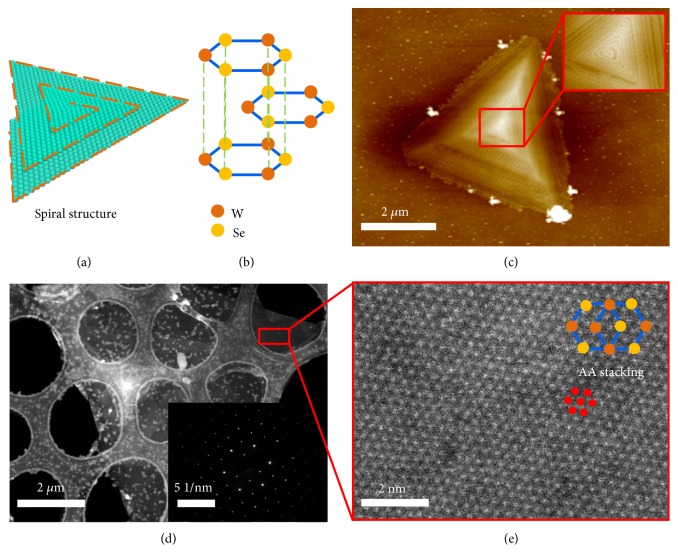
**Structure Characterizations of 3D spiral WSe**_**2**_. (a, b) Schematic illustration of a spiral WSe_2_ structure (a) and the corresponding basal planes stacking order (b). (c) AFM image of a WSe_2_ flake. Scale bar: 2 *μ*m. Inset: high-resolution AFM image of the region marked with red box. (d) STEM image of a typical spiral WSe_2_. Scale bar: 2 *μ*m. Inset: the corresponding SAED pattern. (e) HAADF image of the region marked with red box shown in (d). Scale bar: 2 nm.

Tungsten diselenide (WSe_2_) was selected as the model TMDC material for constructing the hybrid 3D spiral TMDC plasmonic structure due to its large second-order nonlinear susceptibility [10–13].  Figure 1(a) shows a typical 3D spiral multilayer structure of WSe_2_, where the basal planes spiral up with gradually decreasing size. Owing to the AA lattice stacking structure (Figure 1(b)), the second-order nonlinear polarization of the adjacent layers would have the same orientation under the linearly polarized laser excitation [14, 15]. This would lead to the constructive interference between them, which is conducive for efficient SH radiation. In addition, compared with the atomic thickness of monolayer WSe_2_, the 3D spiral WSe_2_ flakes with the increase of thickness have much longer light-matter interaction length [16–18], which is beneficial for the enhancement of light-matter interaction and thus the enhanced SHG.

The 3D spiral WSe_2_ nanostructures were fabricated with an atmospheric pressure chemical vapor deposition method (Fig. [Sec supplementary-material-1]; see Materials and Methods) [19–21]. As shown in Figure 1(c), the atomic force microscopy image (AFM) reveals that the as-prepared WSe_2_ nanostructure has well-defined 2D plate-like triangle morphology with tetrahedral structure. The magnified AFM image (inset of Figure 1(c)) shows that the basal planes of 3D WSe_2_ flake shrink gradually from the bottom to the top layers with a clear 3D spiral structure, indicating the broken inversion symmetry of WSe_2_ [22, 23]. The scanning transmission electron microscopy (STEM) image of a typical WSe_2_ nanostructure (Figure 1(d)) and corresponding selected area electron diffraction (SAED) indicate the single crystalline nature of the WSe_2_ flake and the good alignment of all the layers [16, 24]. Moreover, the atomic resolution STEM high-angle annular dark-field image (HAADF) (Figure 1(e)) clearly shows that the as-prepared WSe_2_ flake has AA stacking configuration of basal planes [25–27], which is beneficial for achieving constructive interference of second-order nonlinear polarization between them.

**Figure 2:**
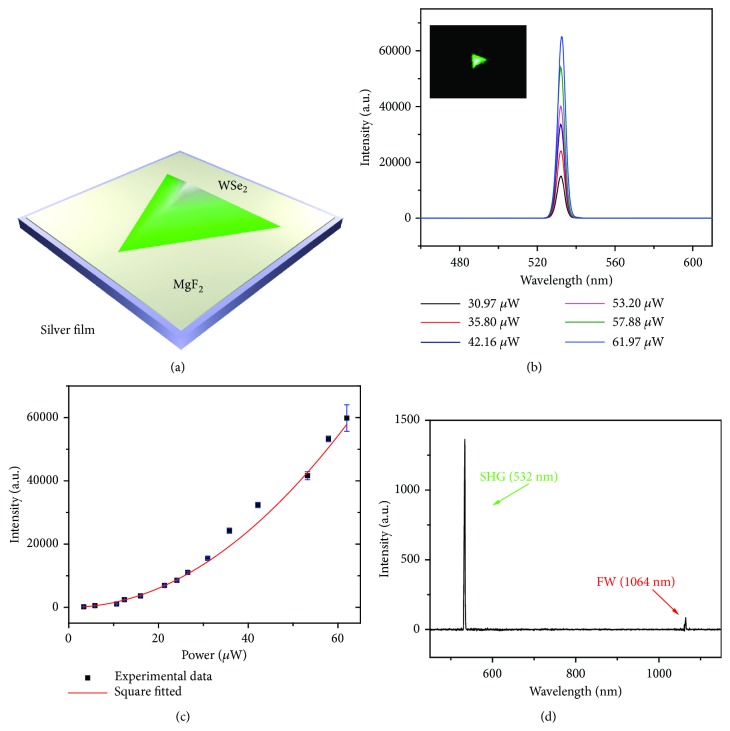
**Hybrid 3D spiral WSe**_**2**_ **plasmonic structure for highly efficient SHG**. (a) Schematic illustration of a hybrid spiral WSe_2_ plasmonic structure where a single WSe_2_ sits on top of the MgF_2_ layer near the Ag film. (b) Spatial resolved spectra collected from the hybrid spiral WSe_2_ structure shown in the inset. Inset: SH image of a single hybrid spiral WSe_2_ structure excited with a CW laser (1064 nm). (c) Measured SHG intensity as a function of FW laser power, which fits to a square dependence. (d) Spectra of SHG and FW from hybrid spiral WSe_2_ plasmonic structure excited with 1064 nm CW laser.

These as-fabricated 3D spiral WSe_2_ structures with broken inversion symmetry provide a promising structure to produce large effective conversion coefficients of second-order nonlinear optical parametric processes. Unfortunately, the thicknesses of these structures are below the diffraction limit (Fig. [Sec supplementary-material-1]), which leads to the poor electric field confinement [28]. Hybrid plasmonic structures, where the dielectric materials are separated from the metallic surface by a nanometer-scale insulating gap, can tightly confine electric field at subwavelength scale due to the excellent field confinement ability of SPPs [29, 30]. This offers an opportunity to enhance the light-matter interaction at nanoscale size. Thus, as illustrated in Figure 2(a), the 3D spiral WSe_2_ structures were transferred onto the top of a smooth silver film with a 10-nanometer magnesium fluoride (MgF_2_) insulating gap to construct hybrid 3D spiral WSe_2_ plasmonic structures [18], which enable the subwavelength optical confinement and low optical losses.

Then, the second-order nonlinear optical response measurements were performed on a home-built far-field micro optical system (Fig. [Sec supplementary-material-1]). A 1064 nm continuous-wave (CW) laser beam was focused on the center of a typical hybrid 3D spiral WSe_2_ plasmonic structure. As demonstrated in the inset of Figure 2(b), a strong green light radiated from the spiral WSe_2_ flake under the FW (1064 nm) excitation, indicating the efficient SH radiation. The spatial resolved spectra taken from spiral WSe_2_ (Figure 2(b)) show that the signals exhibit narrow peaks centered at 532 nm, which is twice the frequency of the FW at 1064 nm. This is consistent with the characteristics of SHG. Power-dependent measurements, shown in Figure 2(c) (the corresponding values are listed in [Sec supplementary-material-1]), demonstrate that the signal intensity has a quadratic power dependence on the FW intensity, further confirming the second-order nature of the emitted light. Furthermore, the polarization-resolved SHG of an individual spiral WSe_2_ flake shown in Fig. [Sec supplementary-material-1] (measured under the parallel polarization configuration (Fig. [Sec supplementary-material-1])) shows a sixfold anisotropic pattern, which agrees well with previous works [31–33]. This suggests a typical threefold rotational symmetry of spiral WSe_2_ crystal, which further confirms the AA stacking mode of WSe_2_ basal planes.

A simple experiment was carried out to estimate the SHG conversion efficiency of the hybrid spiral WSe_2_ plasmonic structure by comparing the intensities of SHG and FW [34, 35]. Figure 2(d) shows the spectra of the reflected SHG (532 nm) and the corresponding pumping laser (1064 nm). According to the optical measurement setup (Fig. [Sec supplementary-material-1]), the conversion efficiency (*η*) of SHG in hybrid 3D spiral WSe_2_ plasmonic structure was estimated according to the following equation:

(1) $$\begin{array}{c} \eta =\frac{Q_{FW}}{Q_{SHG}}\cdot \frac{T_{FW\left(obj\right)}}{T_{SHG\left(obj\right)}}\cdot \frac{T_{FW\left(DM\right)}}{T_{SHG\left(DM\right)}}\cdot \frac{T_{FW\left(SP\right)}}{T_{SHG\left(SP\right)}}\cdot \frac{I_{SHG}}{I_{FW}} \end{array}$$

where Q_FW_ and Q_SHG_ correspond to the collective efficiency of spectrometer for the wavelength of FW and SHG; T_FW(obj)_ and T_SHG(obj)_ represent the transmittance of objective for FW and SHG; and T_FW(DM)_ (T_SHG(DM)_) and T_FW(SP)_ (T_SHG(SP)_) are transmittance of the short-pass dichroic mirror and short-pass filter for the wavelength of FW (SHG), respectively. For statistical analysis, I_FW_ and I_SHG_ are the intensities of FW and SHG obtained from the measured spectra shown in Figure 2(d) and Fig. [Sec supplementary-material-1]. The average ratio of I_SHG_/I_FW_ is listed in [Sec supplementary-material-1]. Moreover, all of the above-mentioned values are demonstrated in [Sec supplementary-material-1]. According to the equation, the normalized SHG conversion efficiency is estimated to be 2.437×10^−5^ under the 1064 nm laser excitation, which is larger than most reported nanostructures ([Sec supplementary-material-1]) [35–41]. This can be partially ascribed to the large second-order nonlinear susceptibility of WSe_2_ (on the order of 10^−7^ m/V).

**Figure 3:**
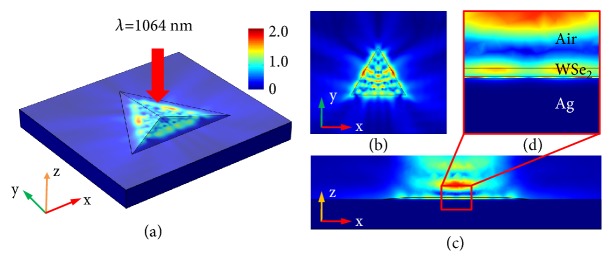
**Simulated electric field distributions in hybrid spiral WSe**_**2**_ **plasmonic structure.** (a) Simulated electric field distributions in hybrid spiral WSe_2_ plasmonic structure. (b, c) The corresponding xy plane (b) and xz plane (c) obtained from the result shown in (a). (d) The magnified image of the region marked with red box shown in (c).

To look deep into the underlying mechanism of extremely high conversion efficiency of SHG in hybrid 3D spiral WSe_2_ plasmonic structure, we carried out a 3D model to simulate the average intensity of the confined electric field excited by FW in hybrid plasmonic structure (see Materials and Methods). For comparison, the electric field distribution of WSe_2_ on SiO_2_/Si substrate was also calculated under the same excitation condition, and all the results were shown in Fig. [Sec supplementary-material-1]. In sharp contrast to the poor electric field confinement of WSe_2_ on the SiO_2_/Si substrate (Fig. [Sec supplementary-material-1]), the hybrid 3D WSe_2_ plasmonic structure shows much more sufficient electric field confinement (Figures 3(a) and 3(b)) [29, 42], which would lead to the enhanced light-matter interaction. Moreover, the images of xz cut-plane, shown in Figures 3(c) and 3(d), reveal the generation of hybrid plasmonic mode under the FW excitation, where the electric field is highly confined in WSe_2_ and the gap region between the flake and metal surface. The small mode area of hybrid plasmonic mode with highly confined electric field enhances the light-matter interaction in hybrid 3D spiral WSe_2_ structure, which is favorable for the enhancement of SHG at nanoscale size [43, 44]. Furthermore, compared with the SiO_2_/Si substrate, the reflection enhancement of silver film due to the intrinsic properties of different materials would further strengthen the light-matter interaction in hybrid 3D spiral WSe_2_ structure, which is also beneficial for obtaining efficient SHG [18, 36]. Thus, in brief, the highly enhanced light-matter interaction resulting from the highly confined electric field and reflection enhancement in hybrid 3D spiral WSe_2_ plasmonic structure, along with the large second-order nonlinear susceptibility of WSe_2_, brings about the extremely high conversion efficiency of SHG.

**Figure 4:**
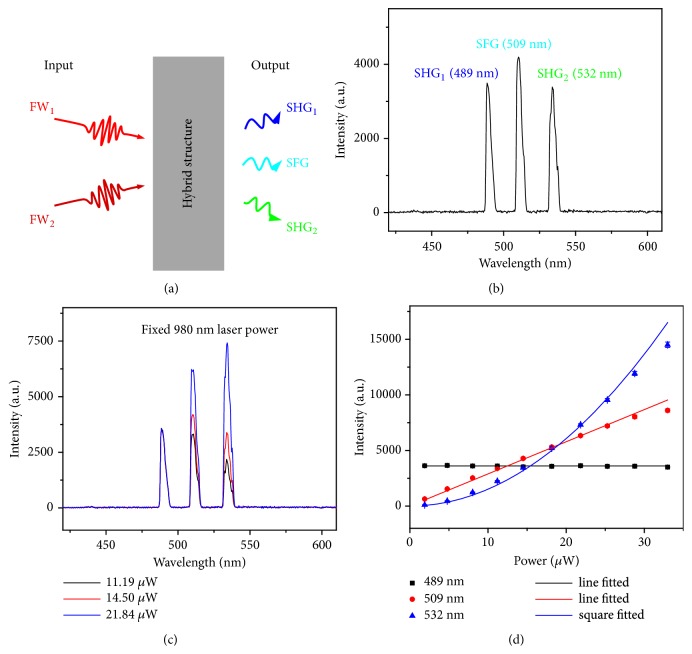
**Simultaneous processes of SHG and SFG in hybrid spiral WSe**_**2**_ **plasmonic structure. **(a) The schematic of simultaneous processes of SHG and SFG. (b) The spectrum collected from the hybrid spiral WSe_2_ plasmonic structure excited with 1064 nm and 980 nm CW laser simultaneously. (c) The spectra collected from the hybrid 3D spiral WSe_2_ plasmonic structure with varied 1064 nm pump power while the 980 nm laser power was fixed. (d) The corresponding signals intensities vary with the increase of the 1064 nm laser power.

Owing to the extremely high second-order nonlinear optical conversion efficiency, the hybrid 3D spiral WSe_2_ plasmonic structure offers a possibility of achieving SHG and SFG simultaneously. Meanwhile, the subwavelength thickness of 3D spiral WSe_2_ would ease the phase-matching condition for second-order nonlinear parametric processes, which also benefits the achievement of SHG and SFG [5, 45]. Under the two FWs with different wavelength (FW_1_ and FW_2_) excitation, the hybrid 3D spiral WSe_2_ plasmonic structure would produce three discrete coherent signals (SHG_1_ of FW_1_, SHG_2_ of FW_2_, and the sum-frequency generation of FW_1_ and FW_2_), as illustrated in Figure 4(a). In our experiments, to obtain efficient SHG and SFG simultaneously, CW lasers with wavelength of 980 nm and 1064 nm were employed as the FWs for the insurance of temporal matching of the pulses. Figure 4(b) demonstrates the spectrum obtained from a typical hybrid 3D spiral WSe_2_ structure under the excitation of FW_1_ and FW_2_. Three discrete sharp peaks with centers at 489 nm, 509 nm, and 532 nm were achieved. Except for the SHG_1_ (489 nm) and SHG_2_ (532 nm) generated from the FW_1_ and FW_2_, respectively, the wavelength centered at 509 nm satisfies the following equation described as

(2) $$\begin{array}{c} \frac{1}{\lambda_{Signal}}=\frac{1}{\lambda_{FW_{1}}}+\frac{1}{\lambda_{FW_{2}}} \end{array}$$

suggesting the efficient SFG in hybrid 3D spiral WSe_2_ plasmonic structures [46].

Power-dependent measurements were further carried out to explore the relationship between SHG and SFG by fixing the power of 980 nm laser. Figures 4(c) and 4(d) (the corresponding values are listed in [Sec supplementary-material-1]) show the dependence of signal intensities on the power of 1064 nm laser. We can see that the intensity of SHG_1_ remains unchanged while those of SFG and SHG_2_ increase with the increase of 1064 nm laser power. In addition, as we can see from Figure 4(d), the intensity of SHG_2_ (532 nm) shows a square dependence of FW_2_ power while that of SFG grows linearly with the increase of FW_2_ power. These results agree well with the mechanism of SHG and SFG, where the intensities of signals varied with the FW power can be expressed as

(3) ISHG2∝A1IFW22

(4) ISFG∝A2IFW1·IFW2

respectively. In addition, similar results were achieved by changing the power of 980 nm laser with fixed 1064 nm laser power (Fig. [Sec supplementary-material-1] and [Sec supplementary-material-1]), further confirming the simultaneous generation of SHG and SFG and the ease of phase-matching condition in hybrid 3D spiral WSe_2_ plasmonic structures.

## 3. Discussion

In summary, highly efficient second-order nonlinear parametric processes were realized in hybrid 3D spiral WSe_2_ plasmonic structures based on the enhanced light-matter interaction through the intense electric field confinement of SPPs. The 3D spiral WSe_2_ with broken inversion symmetry, where the basal planes spiral up from the bottom to the top layers with gradually decreasing size, exhibits large nonvanishing second-order nonlinear susceptibility. The constructive interference of SH fields between the neighboring atomic layers, along with the enhanced light-matter interaction, results in the extremely high SHG conversion efficiency (2.437×10^−5^). Moreover, SHG and SFG were simultaneously achieved due to the enhanced second-order nonlinear processes and the ease of phase-matching condition in hybrid 3D spiral WSe_2_ plasmonic structures. We believe that the results demonstrated here would provide enlightenment for the construction of typical structures for efficient nonlinear processes.

## 4. Materials and Methods

### 4.1. Preparation of 3D Spiral WSe_2_

The tungsten oxide (WO_3_, 99.99%) and selenium powders (Se, 99.99%) were purchased from Sigma Aldrich and used without further treatment. An atmospheric pressure chemical vapor deposition (CVD) method (Fig. [Sec supplementary-material-1]) was carried out to fabricate the 3D spiral WSe_2_ nanostructures [19–21]. The CVD system with two separated heating zones was employed to separately control the evaporation temperatures of WO_3_ and Se powders. The high nucleation rate at the initial stage was applied to produce a dislocation center with high spiraling-up activity [16, 23]. In a typical preparation, 200 mg WO_3_ powders were loaded in a ceramic boat covered with clean face down SiO_2_/Si substrate at the center of heating zone 1. Another ceramic boat containing 600 mg Se powders was placed at the high temperature region close to WO_3_ (heating zone 2). To increase the nucleation rate for the increasing possibility of getting spirals, we set the evaporation temperature of Se power at 400°C. Before heating, the tube was vacuum-pumped to evacuate the air and then refilled with mixture of H_2_/Ar (with 5% H_2_, the carrier gas) to atmospheric pressure. After that, the center of the heating zone 1 (heating zone 2) was heated to 950°C (400°C) in 30 min and held for 15 min. Then, the furnace was cooled to room temperature naturally. During the growth process, 300 sccm mixture of H_2_/Ar is continuously supplied as the carrier gas.

### 4.2. Characterization

The WSe_2_ nanostructures were transferred onto different substrates for the measurements of atomic force microscopy (AFM, Bruker Multimode 8) and aberration-corrected scanning transmission electron microscopy (STEM, JEM-ARM 200F). The optical measurements were carried out on home-built far-field micro optical systems. The schematic demonstration of the experimental setups for optical characterization is shown in [Sec supplementary-material-1]. In a typical SHG measurement, a continuous-wave laser (1064 nm, Spectra Physics) was focused on a single WSe_2_ nanosheet through an objective to obtain SH radiation. The excitation laser (FW) was filtered with a 750 nm short-pass filter. The emission from the WSe_2_ nanostructure was collected with the same objective and recorded with a thermal-electrically cooled CCD (Princeton Instruments, ProEm: 1600B). For SHG and SFG measurements, two continuous-wave lasers with wavelength of 1064 nm and 980 nm were employed as the FWs and focused onto a typical WSe_2_ nanosheet through the same objective to achieve SHG and SFG simultaneously. The generated signals were also collected with the same objective and recorded with the thermal-electrically cooled CCD.

### 4.3. Numerical Simulation

The numerical simulations were carried out with the commercial software COMSOL, which can solve three-dimensional Maxwell equations by the finite element method. The frequency domain Wave Optics module was employed. A tetrahedral solid structure was utilized to model WSe_2_ structure with a refractive index (n) of 3.5, sitting on top of 1 *μ*m Ag film with 10 nm MgF_2_ layer (n=1.38). A linearly polarized beam with wavelength of 1064 nm was employed to irradiate from the top of hybrid 3D spiral WSe_2_ plasmonic structure. For hybrid plasmonic structure, the permittivity of Ag is -57.906+0.60878i at wavelength of 1064 nm.

## Data Availability

All data needed to evaluate the conclusions in the paper are present in the paper and/or the Supplementary Materials. Additional data related to this paper may be requested from the authors.

## Supplementary Materials

Supplementary Materials See the attached pdf file for Supplementary Materials, which includes the following: Fig. S1. Schematic illustration of the CVD system for WSe_2_ nanostructures growth. Fig. S2. Height profile of the WSe_2_ nanostructure shown in Figure 1(c). Fig. S3. Schematic demonstration of the experimental setups for optical characterization. Fig. S4. Polarization dependent SHG in hybrid 3D spiral WSe_2_ plasmonic structure measured under the parallel polarization configuration. Fig. S5. Spectra of SHG and FW obtained from three typical hybrid spiral WSe_2_ plasmonic structures excited with 1064 nm CW laser of varied powers. Fig. S6. Simulated electric field distribution in 3D spiral WSe_2_ on SiO_2_/Si substrate. Fig. S7. The spectra collected from the hybrid 3D spiral WSe_2_ plasmonic structure with varied 980 nm pump laser power while the 1064 nm laser power was fixed. Table S1. The corresponding mean values and standard error of I_SHG_ at varied powers of 1064 nm laser shown in Figure 2(c). Table S2. The values of I_SHG_ and I_FW_ shown in Fig. S5 and the corresponding I_SHG_/I_FW_. Table S3. The ratio values for the simple estimation of SHG conversion efficiency. Table S4. The conversion efficiencies of SHG in different micro-/nanostructures. Table S5. The corresponding mean values and standard error of I_SHG_ and I_SFG_ at varied powers of 1064 nm laser when the power of 980 nm laser was fixed, shown in Figure 4(d). Table S6. The corresponding mean values and standard error of I_SHG_ and I_SFG_ at varied powers of 980 nm laser when the power of 1064 nm laser was fixed, shown in Fig. S7B. References [31–33].
