# Supplementary material for: Hybrid Three-Dimensional Spiral WSe2 Plasmonic Structures for Highly Efficient Second-Order Nonlinear Parametric Processes
Source: Research (Wash D C). 2018 Dec 9;2018:4164029. doi: 10.1155/2018/4164029 (PMC6750081; doi:10.1155/2018/4164029)
Supplement: Supplementary Materials — See the attached pdf file for Supplementary Materials, which includes the following: Fig. S1. Schematic illustration of the CVD system for WSe2 nanostructures growth. Fig. S2. Height profile of the WSe2 nanostructure shown in Figure 1(c). Fig. S3. Schematic demonstration of the experimental setups for optical characterization. Fig. S4. Polarization dependent SHG in hybrid 3D spiral WSe2 plasmonic structure measured under the parallel polarization configuration. Fig. S5. Spectra of SHG and FW obtained from three typical hybrid spiral WSe2 plasmonic structures excited with 1064 nm CW laser of varied powers. Fig. S6. Simulated electric field distribution in 3D spiral WSe2 on SiO2/Si substrate. Fig. S7. The spectra collected from the hybrid 3D spiral WSe2 plasmonic structure with varied 980 nm pump laser power while the 1064 nm laser power was fixed. Table S1. The corresponding mean values and standard error of ISHG at varied powers of 1064 nm laser shown in Figure 2(c). Table S2. The values of ISHG and IFW shown in Fig. S5 and the corresponding ISHG/IFW. Table S3. The ratio values for the simple estimation of SHG conversion efficiency. Table S4. The conversion efficiencies of SHG in different micro-/nanostructures. Table S5. The corresponding mean values and standard error of ISHG and ISFG at varied powers of 1064 nm laser when the power of 980 nm laser was fixed, shown in Figure 4(d). Table S6. The corresponding mean values and standard error of ISHG and ISFG at varied powers of 980 nm laser when the power of 1064 nm laser was fixed, shown in Fig. S7B. References [31–33]. [file 4164029.f1.pdf]

Supplementary Materials for

Hybrid Three-Dimensional Spiral WSe_2 Plasmonic Structures for Highly Efficient Second Order Nonlinear Parametric Processes

Xianqing Lin, Yingying Liu, Kang Wang, Xiaolong Liu, Yongli Yan, Yong Jun Li, Jiannian Yao, and Yong Sheng Zhao

This PDF file includes:

Fig. S1. Schematic illustration of the CVD system for WSe_2 nanostructures growth.

Fig. S2. Height profile of the WSe_2 nanostructure shown in Fig. 1C.

Fig. S3. Schematic demonstration of the experimental setups for optical characterization.

Fig. S4. Polarization dependent SHG in hybrid 3D spiral WSe_2 plasmonic structure measured under the parallel polarization configuration.

Fig. S5. Spectra of SHG and FW obtained from three typical hybrid spiral WSe_2 plasmonic structures excited with 1064 nm CW laser of varied powers.

Fig. S6. Simulated electric field distribution in 3D spiral WSe_2 on SiO_2/Si substrate.

Fig. S7. The spectra collected from the hybrid 3D spiral WSe_2 plasmonic structure with varied 980 nm pump laser power while the 1064 nm laser power was fixed.

Table S1. The corresponding mean values and standard error of I_{SHG} at varied powers of 1064 nm laser shown in Fig. 2C.

Table S2. The values of I_{SHG} and I_{FW} shown in Fig. S5 and the corresponding I_{SHG}/I_{FW}.

Table S3. The ratio values for the simple estimation of SHG conversion efficiency**.**

Table S4. The conversion efficiencies of SHG in different micro-/nanostructures.

Table S5. The corresponding mean values and standard error of I_{SHG} and I_{SFG} at varied powers of 1064 nm laser when the power of 980 nm laser was fixed shown in Fig. 4D.

Table S6. The corresponding mean values and standard error of I_{SHG} and I_{SFG} at varied powers of 980 nm laser when the power of 1064 nm laser was fixed shown in Fig. S7B.

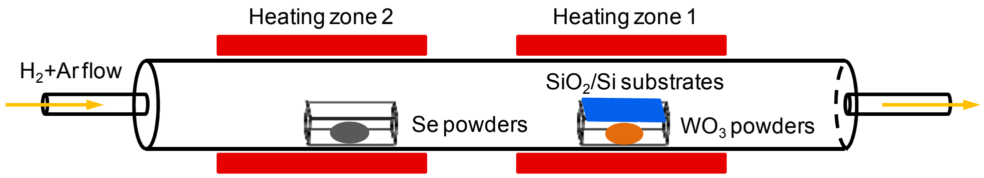

Fig. S1. Schematic illustration of the CVD system for WSe_2 nanostructures growth.

As demonstrated in Fig. S1, a CVD system with two separated heating zones was employed, so that the evaporation temperature of WO_3 and Se powders can be separately controlled. Before heating, the tube was vacuum pumped to evacuate the air and then refilled with mixture of H_2/Ar to atmospheric pressure. During the growth process, 300 sccm mixture of H_2/Ar is continuously supplied as the carrier gas.

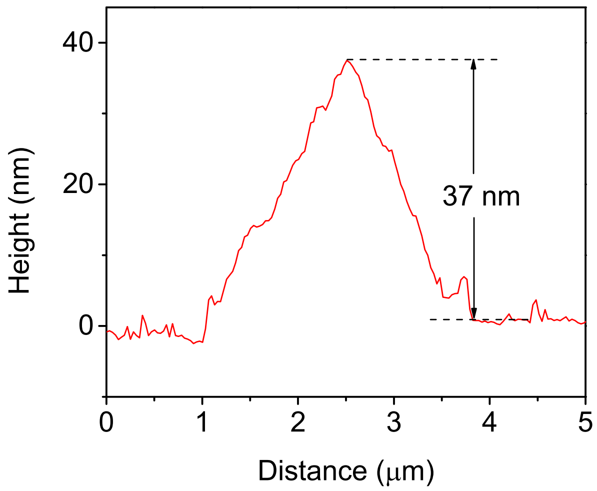

Fig. S2. Height profile of the WSe_2 nanostructure shown in Fig. 1C.

As demonstrated in Fig. S2, the height of 3D spiral WSe_2 nanostructures is about 37 nm, which is far below the diffraction limit of light. This would lead to poor electric field confinement, which is unfavorable for the efficient nonlinear optical parametric processes.

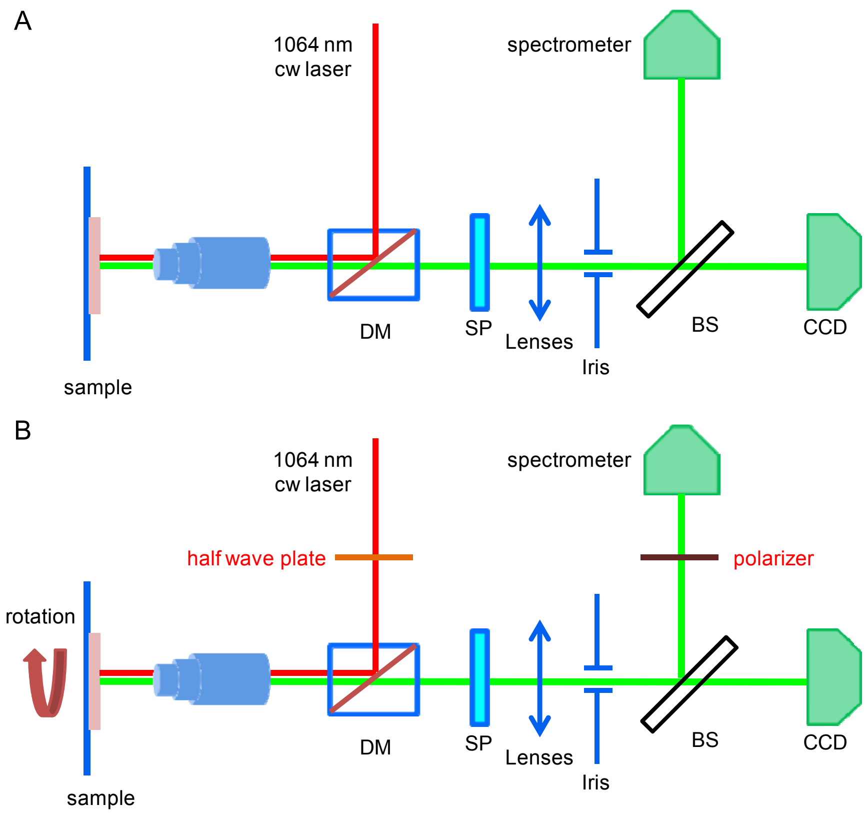

Fig. S3. Schematic demonstration of the experimental setups for optical characterization.

As illustrated in Fig. S3A, the second order nonlinear optical parametric processes measurements were performed in a reflection geometry using vertical incidence excitation. The WSe_2 nanostructures were excited with continuous wave laser (Spectra Physics) focused down to 4 μm diameter spot through an objective (Nikon CFLU Plan, 50x, N.A.=0.8) to obtain SH radiation. The excitation laser (FW) was filtered with a 750 nm short-pass filter. The emission from the WSe_2 nanostructure was dispersed with a grating (150 G/mm) and recorded with a thermal-electrically cooled CCD (Princeton Instruments, ProEm: 1600B). For polarization dependent SHG measurements (parallel polarization configuration), as shown in Fig. S3B, the polarization of FW signals was altered with a half wave plate. A polarizer with a fixed polarization was placed in front of spectrometer to select the parallel components of the SH radiation with respect to the polarization of FW. In this setup, the samples were freely rotated to obtain the orientational dependence of the SH response.

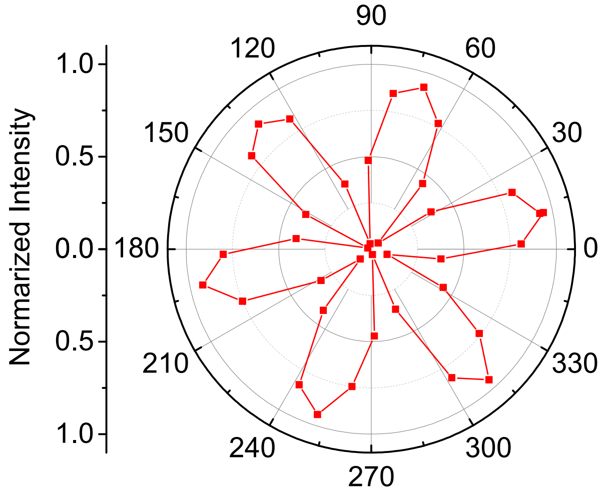

Fig. S4. Polarization dependent SHG in hybrid 3D spiral WSe_2 plasmonic structure measured under the parallel polarization configuration.

As plotted in Fig. S4, the polarization dependent SHG of an individual spiral WSe_2 flake shows a six-fold anisotropic pattern, which agrees well with previous works (*31-33*). This suggests a typical three-fold rotational symmetry of spiral WSe_2 crystal with AA lattice stacking mode, which is beneficial for achieving efficient SH radiation.

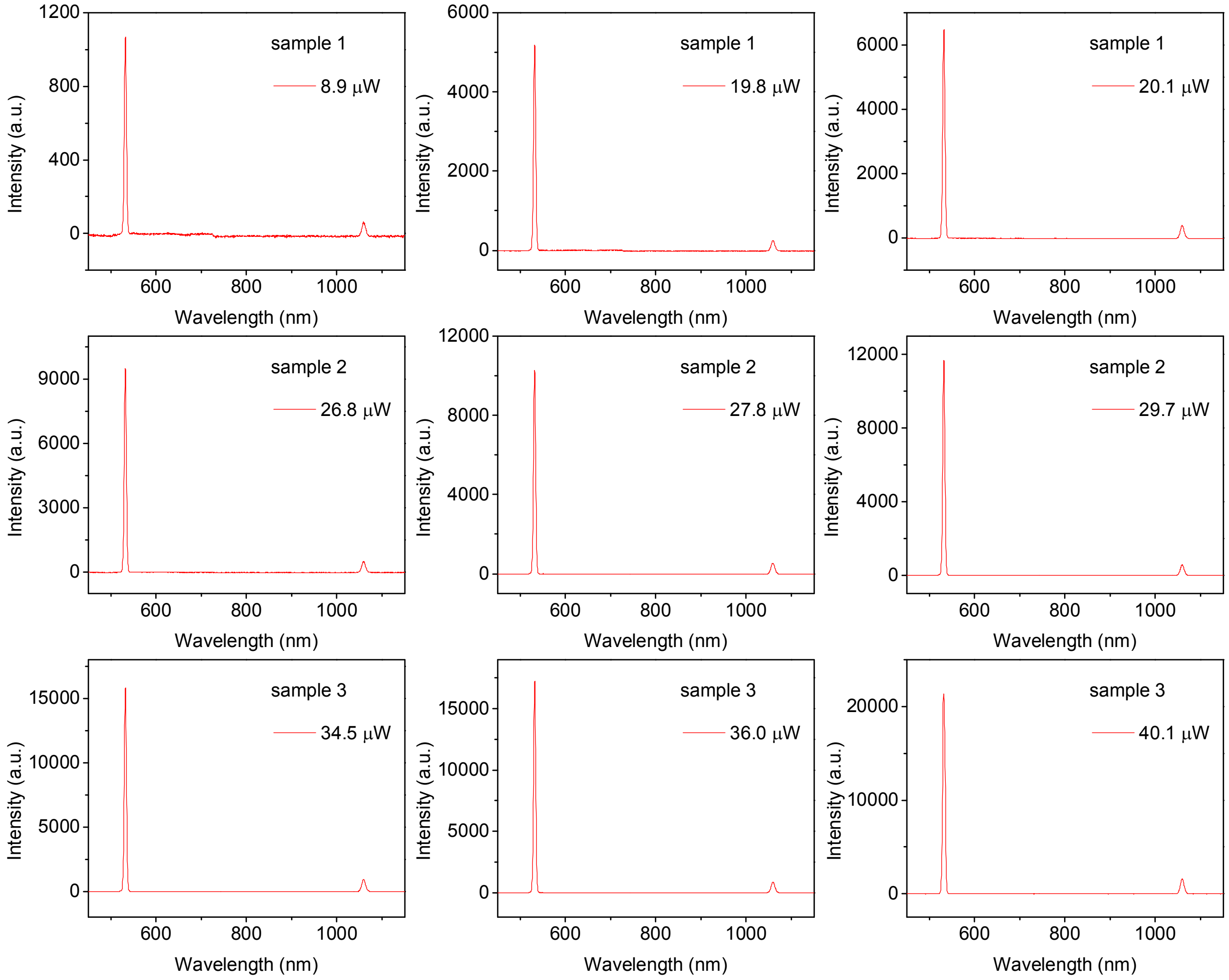

Fig. S5. Spectra of SHG and FW obtained from three typical hybrid spiral WSe_2 plasmonic structures excited with 1064 nm CW laser of varied powers.

As we can see from Fig. S5, to eliminate the influence brought by the different samples and excitation laser powers, we excited three typical hybrid spiral WSe_2 plasmonic structures with 1064 CW laser of varied powers. The corresponding intensities of SHG and FW were listed in Table S2. The average ratio of I_{SHG}/I_{FW} is about 18.13. Thus, according to the ratio values demonstrated in Table S3, we can estimate that the SHG conversion efficiency is about 2.437×10^{-5}, which is larger than most reported nanostructures (Table S4). This indicates the great potential of hybrid 3D spiral plasmonic structures for highly efficient second-order nonlinear parametric processes.

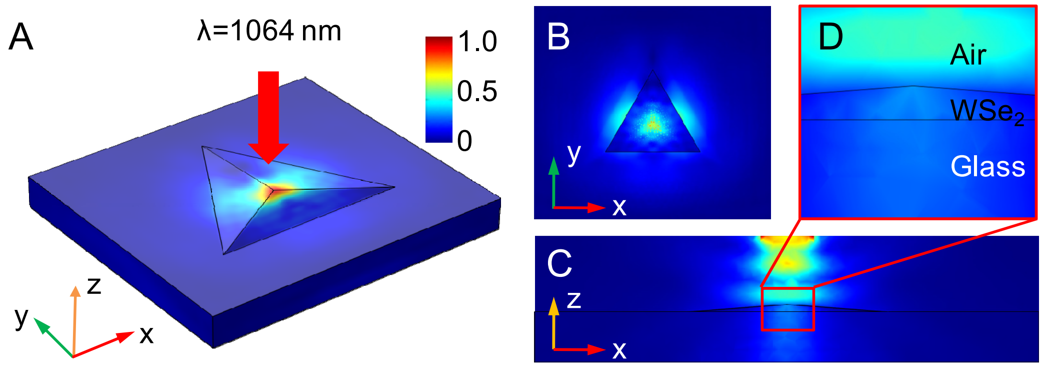

Fig. S6. Simulated electric field distribution in 3D spiral WSe_2 on SiO_2/Si substrate. (**A**) Simulated electric field distributions in 3D spiral WSe_2 on SiO_2/Si substrate. (**B**, **C**) The corresponding xy plane (B) and xz plane (C) obtained from the result shown in (A). (**D**) The magnified image of the region marked with red box shown in (C).

Fig. S6 demonstrates the electric field distribution in 3D spiral WSe_2 on SiO_2/Si substrate under the excitation of FW (1064 nm). As we can see, poor electric field confinement was obtained due to the small sizes of WSe_2 (at subwavelength scale), which leads to weak light-matter interaction. As a result, the SHG conversion efficiency is limited in 3D spiral WSe_2 on SiO_2/Si substrate in spite of its large second-order nonlinear susceptibility.

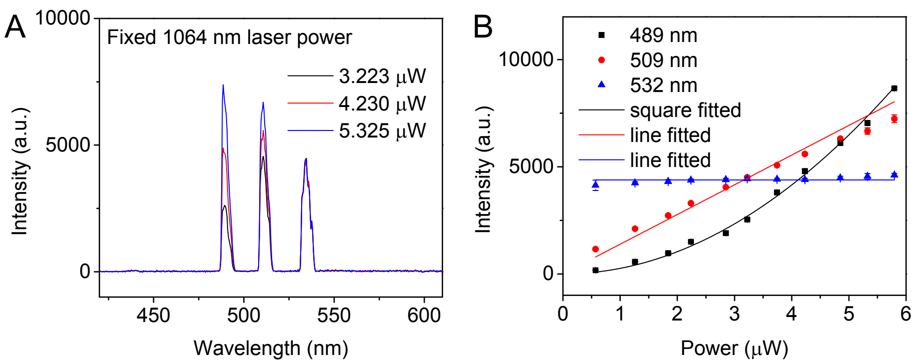

Fig. S7. (**A**) The spectra collected from the hybrid 3D spiral WSe_2 plasmonic structure with varied 980 nm pump laser power while the 1064 nm laser power was fixed. (**B**) The corresponding signals intensities vary with the increase of the 980 nm laser power.

As shown in Fig. S7, the intensity of SHG_2 remains unchanged while the intensities of SFG and SHG_1 increase with the increase of 1064 nm laser power. Moreover, the intensity of SHG_1 (489 nm) shows a square dependence of FW_1 power while that of SFG grows linearly with the increase of FW_2 power, which is in consistence with that shown in Fig. 4 (C and D), indicating the simultaneous generation of SHG and SFG.

Table S1. The corresponding mean values and standard error of I_{SHG} at varied powers of 1064 nm laser shown in Fig. 2C

Power (μW)	Mean	Standard error
3.354	189.8	13.29
5.827	554.2	34.29
10.67	1069.3	45.92
12.40	2425.8	62.78
15.99	3618.7	61.41
21.33	6915.0	112.10
24.10	8551.0	196.04
26.52	11066.7	242.66
30.97	15511.8	398.64
35.80	24244.5	849.25
42.16	32357.3	841.90
53.20	41621.0	1254.99
57.88	53284.8	893.10
61.97	59850.8	4208.03

As we can see from the results of power dependent SHG intensities listed in Table S1 (corresponding to the data in Fig. 2C), the standard errors of the corresponding wavelength are relatively small when compared with the mean values. Similar results were obtained in the simultaneous generation of SHG and SFG (Table S5 and S6). This can be ascribed to the steady output power of the continuous wave lasers, which is conducive to obtain the precise power dependence measurements of SHG and SFG intensities.

Table S2. The values of I_{SHG} and I_{FW} shown in Fig. S5 and the corresponding I_{SHG}/I_{FW}

Spectrum	I_{SHG}	I_{FW}	I_{SHG}/I_{FW}
1	1060	59	17.97
2	5180	249	20.80
3	6463	388	16.66
4	9493	486	19.53
5	10268	535	19.19
6	11689	560	20.87
7	15767	919	17.16
8	17198	874	19.68
9	21244	1585	13.40
Fig. 2D	1364	85	16.04
Average ratio			18.13

Table S3. **The ratio values for the simple estimation of SHG conversion efficiency**

Q_{FW}/Q_{SHG}	$T_{FW(DM)}/T_{SHG(DM)}$	$T_{FW(SP)}/T_{SHG(SP)}$	$T_{FW(obj)}/T_{SHG(obj)}$	I_{SHG}/I_{FW}
2/90	1.04/97.5	0.58/80.7	70.3/89.1	18.13

Table S4. **The conversion efficiencies of SHG in different micro-/nanostructures**

Material	Size	Conversion efficiency	Reference
GaAs	5 μm	5×10^{-5}	*Nat. Commun.* **5**, 3109 (2014**)**
CdTe	800 nm	10^{-10}	*Nano Lett.* **16**, 4807-4810 (2016)
GaP	150 nm	2×10^{-9}	*Nano Lett.* **12**, 820-826 (2012)
CdS/Au	1 μm	2×10^{-8}	*ACS Nano* **9**, 5018-5026 (2015)
CdS/Ag	230 nm	3×10^{-6}	*Nat. Commun.* **5**, 5432 (2014)
Silica/Au	120 nm	1.8×10^{-9}	*Nano Lett.* **11**, 5519-5523 (2011)
Au	1 μm	1.25×10^{-9}	*Nano Lett.* **12**, 4997-5002 (2012)

Table S5. **The corresponding mean values and standard error of I_{SHG} and I_{SFG} at varied powers of 1064 nm laser when the power of 980 nm laser was fixed shown in Fig. 4D**

Power (μW)	Mean (489 nm)	Standard error	Mean (509 nm)	Standard error	Mean (532 nm)	Standard error
1.892	3627.5	39.74	637.7	18.65	104.3	18.37
4.780	3656.2	32.07	1541.3	44.20	450.3	27.07
8.028	3610.5	45.53	2531.8	56.95	1227.8	34.01
11.19	3601.5	34.96	3391.0	71.79	2236.2	63.46
14.50	3524.2	75.64	4286.2	101.06	3443.3	51.90
18.15	3576.7	70.63	5307.2	124.09	5202.3	122.08
21.84	3629.8	52.02	6334.5	105.07	7311.5	82.77
25.27	3573.7	38.13	7207.2	119.02	9543.7	124.57
28.77	3584.2	45.46	8030.7	147.76	11924.8	179.74
32.98	3505.7	67.62	8601.8	149.42	14503.5	247.35

Table S6. The corresponding mean values and standard error of I_{SHG} and I_{SFG} at varied powers of 980 nm laser when the power of 1064 nm laser was fixed shown in Fig. S7B

Power (μW)	Mean (489 nm)	Standard error	Mean (509 nm)	Standard error	Mean (532 nm)	Standard error
0.570	172.2	11.62	1157.7	23.50	4135.8	242.99
1.261	564.8	17.93	2110.8	47.78	4245.7	50.09
1.840	964.5	30.63	2730.8	39.16	4315.3	89.11
2.238	1500.8	33.10	3299.5	50.87	4376.0	52.59
2.848	1903.2	52.13	4049.5	46.78	4397.5	65.90
3.223	2538.2	26.83	4505.4	77.10	4440.6	72.77
3.740	3803.5	36.14	5072.0	53.76	4414.8	48.44
4.230	4797.5	89.93	5597.8	74.01	4398.8	62.37
4.850	6109.7	92.54	6312.0	66.13	4474.8	87.36
5.325	7042.8	117.48	6668.2	155.12	4549.0	137.44
5.795	8663.3	46.79	7243.8	182.27	4609.8	81.90
